# Bacteria-Oil Microaggregates Are an Important Mechanism for Hydrocarbon Degradation in the Marine Water Column

**DOI:** 10.1128/mSystems.01105-21

**Published:** 2021-10-05

**Authors:** Amanda M. Achberger, Shawn M. Doyle, Makeda I. Mills, Charles P. Holmes, Antonietta Quigg, Jason B. Sylvan

**Affiliations:** a Department of Oceanography, Texas A&M University, College Station, Texas, USA; b Department of Marine Biology, Texas A&M University-Galveston, Galveston, Texas, USA; ExxonMobil Research and Engineering

**Keywords:** marine oil snow, microbial aggregate, hydrocarbon degradation

## Abstract

Following oil spills in aquatic environments, oil-associated flocculants observed within contaminated waters ultimately lead to the sedimentation of oil as marine oil snow (MOS). To better understand the role of aggregates in hydrocarbon degradation and transport, we experimentally produced a MOS sedimentation event using Gulf of Mexico coastal waters amended with oil or oil plus dispersant. In addition to the formation of MOS, smaller micrometer-scale (10- to 150-μm) microbial aggregates were observed. Visual inspection of these microaggregates revealed that they were most abundant in the oil-amended treatments and frequently associated with oil droplets, linking their formation to the presence of oil. The peak abundance of the microaggregates coincided with the maximum rates of biological hydrocarbon oxidation estimated by the mineralization of ^14^C-labeled hexadecane and naphthalene. To elucidate the potential of microaggregates to serve as hot spots for hydrocarbon degradation, we characterized the free-living and aggregate-associated microbial assemblages using 16S rRNA gene sequencing. The microaggregate population was found to be bacterially dominated and enriched with putative hydrocarbon-degrading taxa. Direct observation of some of these taxa using catalyzed reporter deposition fluorescence *in situ* hybridization confirmed their greater abundance within microaggregates relative to the surrounding seawater. Metagenomic sequencing of these bacteria-oil microaggregates (BOMAs) further supported their community’s capacity to utilize a wide variety of hydrocarbon compounds. Taken together, these data highlight that BOMAs are inherent features in the biological response to oil spills and likely important hot spots for hydrocarbon oxidation in the ocean.

**IMPORTANCE** Vast quantities of oil-associated marine snow (MOS) formed in the water column as part of the natural biological response to the Deepwater Horizon drilling accident. Despite the scale of the event, uncertainty remains about the mechanisms controlling MOS formation and its impact on the environment. In addition to MOS, we observed micrometer-scale (10- to 150-μm) aggregates whose abundance coincided with maximum rates of hydrocarbon degradation and whose composition was dominated by hydrocarbon-degrading bacteria with the genetic potential to metabolize a range of these compounds. This targeted study examining the role of these bacteria-oil microaggregates in hydrocarbon degradation reveals details of this fundamental component of the biological response to oil spills, and with it, alterations to biogeochemical cycling in the ocean.

## INTRODUCTION

Forecasting the fate of marine oil spills depends on a mechanistic understanding of the various oil biodegradation and transport processes which occur in the ocean. The role of microorganisms in marine oil spill response has been a topic of major research in the last decade (1–6). Such work has yielded significant progress in determining the composition, function, and activity of the water column microbial community involved in hydrocarbon pollution remediation. A few such studies have noted the presence of small clusters of aggregated cells with diameters of <500 μm, here called microaggregates (1, 7–10). One of these studies found a correlation between oil concentration and microaggregate abundance, linking their formation to the presence of oil (10). Despite their potential importance, little research has been conducted focusing on microaggregates, and thus, their impact on the transport and degradation of hydrocarbons following a spill is largely unknown.

In contrast, the importance of macroscale marine oil snow (MOS) sedimentation and flocculent accumulation (MOSSFA) events (11, 12) in determining the fate of spilled oil has recently garnered much attention. The 2010 Deepwater Horizon (DwH) oil spill in the Gulf of Mexico demonstrated that marine snow can transport enormous amounts of oil to the seafloor through the formation of large (≥500-μm), mucus-rich, oil-associated aggregates (11–14). It is believed that this MOSSFA event was a direct result of the production of exopolymeric substances (EPS) by phytoplankton and bacteria exposed to oil (7, 13). Numerous marine microorganisms have been shown to produce EPS that can serve as biosurfactants that emulsify hydrocarbons and enhance their biodegradability (15, 16). EPS also provide a “sticky” matrix that can promote the aggregation of microorganisms with oil droplets suspended in the water column (1, 13, 17, 18).

This type of biological response was not unique to the DwH spill. Evidence for MOS events was also documented following the 1977 Tsesis oil spill in Sweden (19) and the 1979 Ixtoc I oil spill in the southern Gulf of Mexico (20). Although microaggregates are too small to formally be considered MOS, their correlation with elevated oil concentrations has led some to speculate that they are possible precursors to MOS formation (10). Despite the growing appreciation for the importance of MOS after an oil spill, we still lack a full understanding of what role microaggregates might serve in such a process or how the degradation of oil incorporated into aggregates differs from that of oil dispersed freely into the water column.

To better explain and predict the fate of oil spilled in the ocean, it is critical to understand the mechanisms of microaggregate formation and their significance in the natural bioremediation of hydrocarbon pollution. In this study, we utilized mesocosm experiments to assess the behavior of microaggregates under control conditions (seawater only) and in the presence of oil. Given the widespread use of dispersants for oil spill remediation, which not only alter the size and abundance of suspended oil droplets but may also impact the aggregation process (21–23), we also tested the effects of chemically dispersed oil. We present data on microaggregate formation (counts, size) as well as a description of their microbial community structure using 16S rRNA gene amplicon sequencing and catalyzed reporter deposition fluorescence *in situ* hybridization (CARD-FISH). We further evaluated the microbial community’s potential for hydrocarbon degradation through metagenomic sequence analysis and radiotracer (^14^C-labeled hexadecane and naphthalene) assays. Our results show the establishment of bacterially dominated microaggregate specific communities and a link between their formation and abundance with hydrocarbon degradation. Furthermore, this work reveals that these bacteria-oil microaggregates (BOMAs) are an important mechanism for hydrocarbon degradation and transport in the water column.

## RESULTS

### Microbial degradation of hydrocarbons.

**FIG 1:**
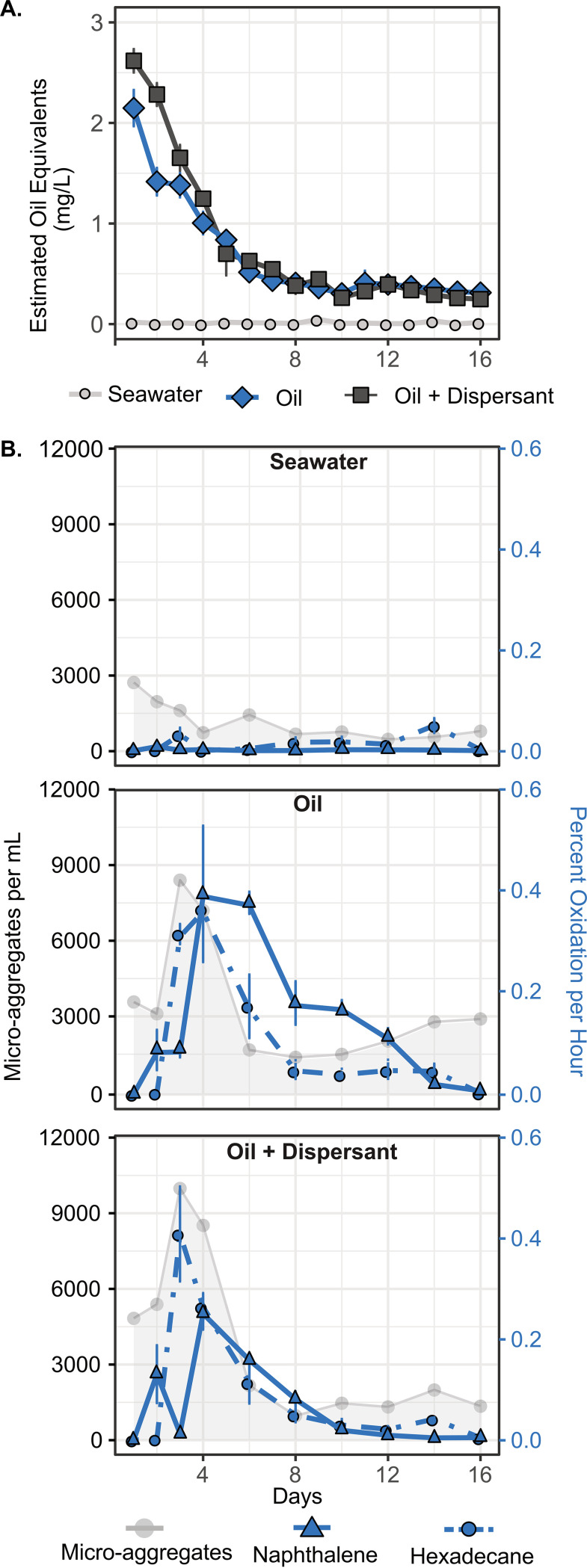
Hydrocarbon degradation over time. (A) Oil concentration expressed as estimated oil equivalents (previously reported by reference 24). (B) Microaggregate abundance is shown as microaggregates per milliliter (left axis). Oxidation rates of [^14^C]hexadecane and [^14^C]naphthalene (right axis), respectively, are shown for each treatment group as a percentage of total available substrate oxidized per hour.

The initial concentration of hydrocarbons in the mesocosms, measured as estimated oil equivalents (EOEs), was statistically the same (*P* value = 0.13) in the oil-only and oil-plus-dispersant treatments: 2.15 (±0.12) and 2.62 (±0.18) mg/liter, respectively (Fig. 1A). This allows us to evaluate the dispersant’s effects on the microaggregates and microbial communities without the confounding influence of variable oil concentrations between treatments. Over the next 8 days, a decrease in the concentration following first-order decay kinetics occurred after which the concentrations remained stable between 0.25 and 0.45 mg/liter for the duration of the experiment (16 days [24]). The decreasing ratios of *n*-C_17_/pristane and *n*-C_18_/phytane over time indicated that a portion of this loss was attributable to biodegradation (see Fig. S1 in the supplemental material) (24). A low but detectable background of hydrocarbons was measured in the seawater control samples that showed no significant change with time (Fig. 1A). Further discussion of individual hydrocarbon components and a detailed account of their degradation rates are presented elsewhere (24).

10.1128/mSystems.01105-21.1 FIG S1 Analysis of oil components as previously described by Shi et al (D. Shi, G. Bera, A. H. Knap, A. Quigg, et al., Mar Pollut Bull 151:110804, 2020, https://doi.org/10.1016/j.marpolbul.2019.110804) showing the ratio of the (A) unresolved complex mixture (UCM) to total resolved (TR) components indicating oil degradation over time and the ratio of (B) *n*-C_17_/pristane and (C) *n*-C_18_/phytane suggesting the degradation of *n*-alkanes was due in part to biological activity. Download FIG S1, EPS file, 1.0 MB. Copyright © 2021 Achberger et al. 2021 Achberger et al. https://creativecommons.org/licenses/by/4.0/ This content is distributed under the terms of the Creative Commons Attribution 4.0 International license.

Cell abundance was similar in all treatments at the start of the experiment (∼6 × 10^5^ cells/ml) and increased to ∼2 × 10^6^ cells/ml after 16 days (Fig. S2). No treatment had statistically different cell concentrations over the entire experiment. Similar trends in cell abundances were noted in previous mesocosm experiments simulating oil spills in surface seawater (10, 25). Microaggregates consisting of tens to hundreds of cells were present in the freshly collected seawater used to establish the mesocosms at *in situ* concentrations of ∼4,400 microaggregates/ml. While the abundance of these microaggregates decreased over time in the seawater control, their abundance in the oil-only and oil-plus-dispersant treatments increased by ≥100% by day 3 before also slowly declining (Fig. 1B). Throughout much of the experiment, more microaggregates were observed in the dispersed oil treatment than in the treatment amended with just oil (Fig. 1B). Within both oil-amended treatments (dispersed and oil-only), >85% of microaggregates examined had visible droplets of oil incorporated into their structure, and eukaryotic cells, such as phytoplankton, were rarely observed. There was no consistent difference in the size distribution of the microaggregates between treatments or over time with the exception of microaggregates in the seawater control after day 12 (Fig. S3).

10.1128/mSystems.01105-21.2 FIG S2 Abundance of total cells in each treatment over time based on direct microscope counts of 4′,6-diamidino-2-phenylindole (DAPI)-stained cells. Download FIG S2, EPS file, 0.7 MB. Copyright © 2021 Achberger et al. 2021 Achberger et al. https://creativecommons.org/licenses/by/4.0/ This content is distributed under the terms of the Creative Commons Attribution 4.0 International license.

10.1128/mSystems.01105-21.3 FIG S3 Size distribution of microaggregates as measured by area. *n* = 100 aggregates per treatment, per time point. Download FIG S3, EPS file, 0.8 MB. Copyright © 2021 Achberger et al. 2021 Achberger et al. https://creativecommons.org/licenses/by/4.0/ This content is distributed under the terms of the Creative Commons Attribution 4.0 International license.

Biological rates of hydrocarbon oxidation were estimated based on the conversion of the C1 carbon of model alkane ([1-^14^C]hexadecane) and polycyclic aromatic hydrocarbon (PAH; [1-^14^C]naphthalene) compounds to [^14^C]carbon dioxide (Fig. 1B). After an initial 24-h lag, hexadecane oxidation rates sharply increased, reaching maximum rates of degradation on day 3 in oil plus dispersant and days 4 to 5 in oil-only treatments. Despite this slight difference in the timing of peak rates, there was no statistical difference in hexadecane oxidation rates between the two oil-amended treatments (*P* ≥ 0.10) over the course of the experiment. In both oil-only and oil-plus-dispersant treatments, naphthalene oxidation rates reached their maximum on day 4. Higher oxidation rates were measured in the oil-only samples than in oil plus dispersant through much of the experiment (days 3, 6, and 10 to 14; *P* < 0.02). Low rates of hexadecane oxidation were measured in the seawater control tanks on day 3 and during the second half of the experiment (days 8 to 14); however, naphthalene oxidation in the seawater control was never above background.

### Microaggregate-associated communities.

Microbial community composition in size-fractionated samples (>3.0 μm and 0.2 to 3.0 μm) was assessed via analysis of 16S rRNA gene amplicon sequencing variants (ASVs). Microscopic examination of size-fractionated samples (*n* = 3) revealed that nearly all microaggregates were retained on the 3.0-μm filters. Therefore, cells on the 3.0-μm filter were considered microaggregate associated, although a small number of free-living cells (<5%) were sometimes also observed to be present in this size fraction via visual inspection under a microscope. A nonmetric multidimensional scaling (NMDS) analysis of community structure revealed that, at the start of the experiment, microaggregate-associated communities (>3.0 μm) were distinct from free-living (0.2 to 3.0 μm) communities regardless of treatment (Fig. S4). Between days 3 and 8, this structure began to noticeably diverge based on treatment such that by the end of the experiment, the communities within treatments were most similar to each other despite size fractionation.

10.1128/mSystems.01105-21.4 FIG S4 Nonmetric multidimensional scaling analysis showing microbial community shifts over time in each treatment as assessed via Bray-Curtis dissimilarity. Download FIG S4, EPS file, 1.4 MB. Copyright © 2021 Achberger et al. 2021 Achberger et al. https://creativecommons.org/licenses/by/4.0/ This content is distributed under the terms of the Creative Commons Attribution 4.0 International license.

**FIG 2:**
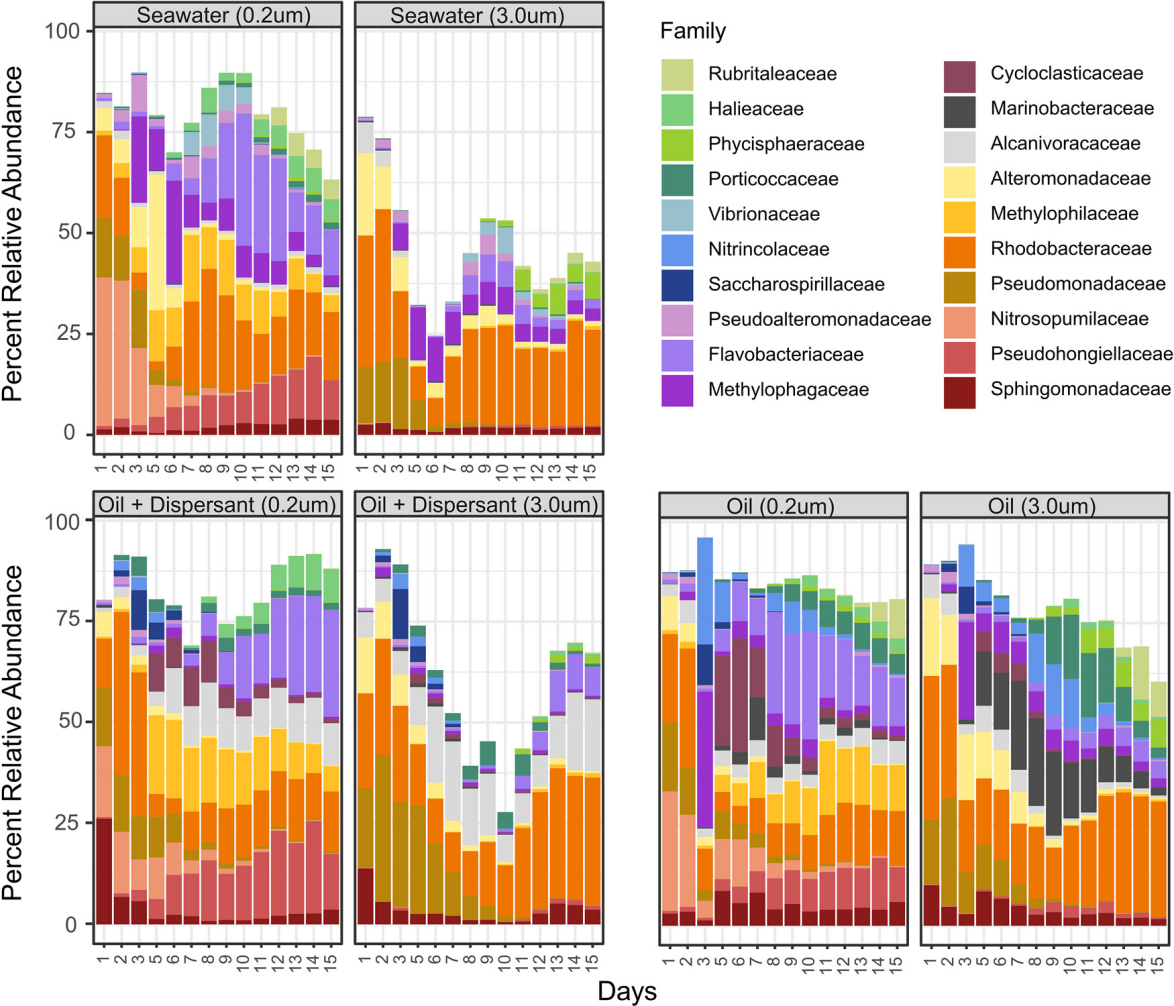
Microbial community composition as assessed via PCR amplicon analysis of the V4 region of the 16S rRNA gene. The community’s taxonomic distribution is described at the family level. Only the 20 most abundant families are shown.

On days 1 and 2, microbial communities in all three treatments were predominantly composed of members of the *Pseudomonadaceae*, *Rhodobacteraceae*, *Alteromonadaceae*, *Alcanivoracaceae*, and *Nitrosopumilaceae* (Fig. 2). By day 3, differences between the two oil-amended treatments and the seawater control were apparent. Compared to the seawater control, an increase in relative abundance by members of the *Nitrincolaceae*, *Saccharospirillaceae*, *Alcanivoracaceae*, and *Cycloclasticaceae* occurred in both the oil-only and oil-plus-dispersant treatments, while *Marinobacteraceae* taxa were prevalent only in the oil-only treatment. By the latter half of the experiment, when hydrocarbons were largely depleted, the relative abundance of *Flavobacteriaceae*, *Phycisphaeraceae*, *Halieaceae*, and *Porticoccaceae* increased in all treatments. Additionally, members of the *Rubritaleaceae* were also present in the seawater control and oil-only treatments. Over the course of the experiment, several of these groups, such as *Cycloclasticaceae*, *Methylophilaceae*, *Pseudohongielliaceae*, *Flavobacteriaceae*, and *Nitrosopumilaceae*, were more abundant on the 0.2- to 3.0-μm size fraction than on the >3.0-μm fraction, revealing significant differences in the microbial community composition of the microaggregate and free-living fractions (Fig. 2).

**FIG 3:**
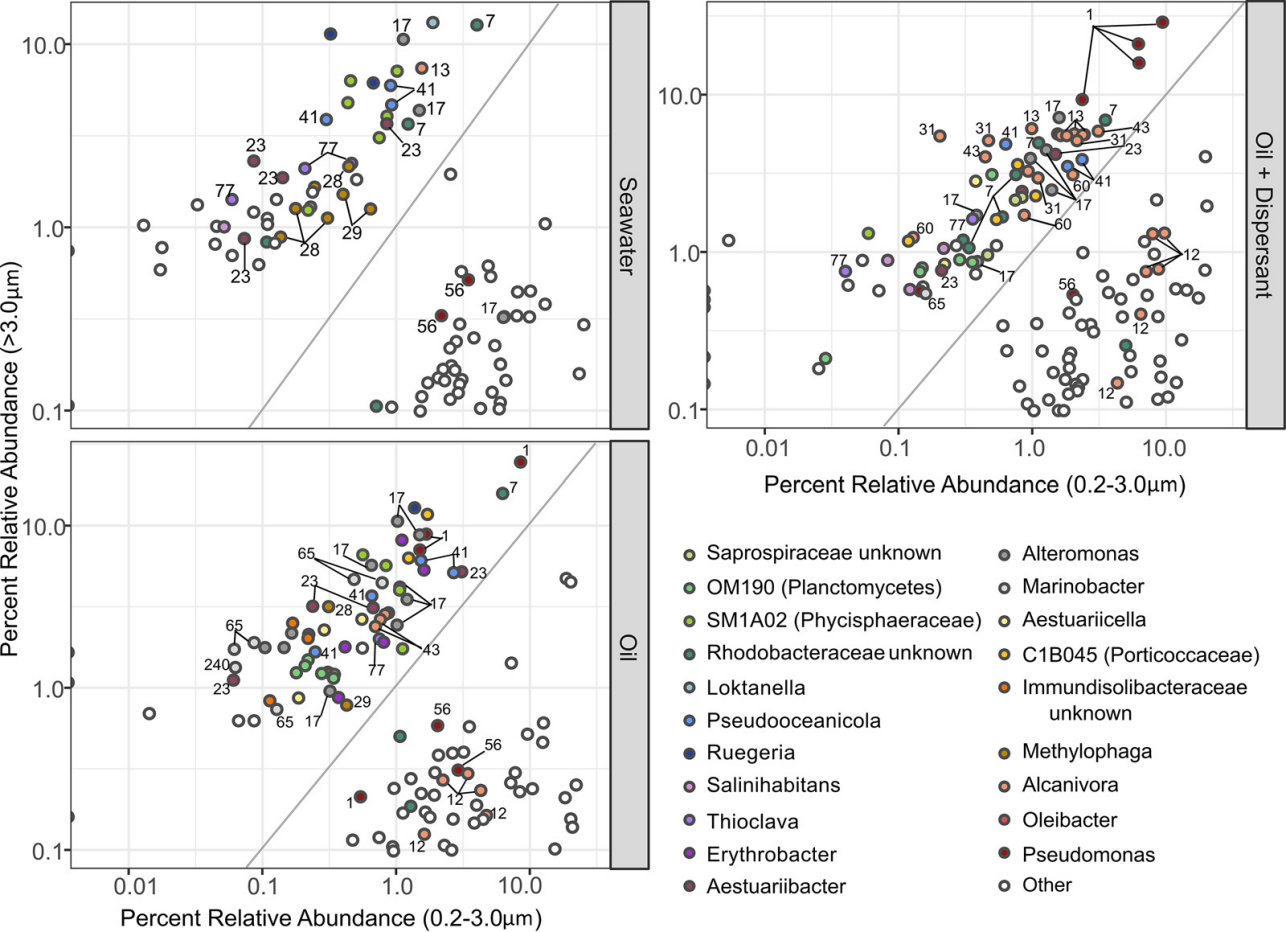
Comparison of the microbial communities between aggregated and free-living groups for each treatment. Differential abundance analysis shows certain phylogenetic groups and ASVs are more abundant in either the >3.0-μm (relative abundance on *y* axis) or 0.2- to 3.0-μm (relative abundance on *x* axis) filter size fraction. The 20 most abundant genera are highlighted with all other less abundant genera as well as groups of unresolved phylogenetic affiliation denoted as other. ASVs of interest discussed in the main text are denoted with numbers within the graphs. A 1:1 ratio between the abundance of ASVs on the >3.0- and 0.2- to 3.0-μm size fractions is depicted as a gray diagonal line within the graphs.

Within each treatment, a linear discriminant analysis (LDA) combined with effect size measurements (26) was used to identify which specific ASVs were statistically more abundant in either the microaggregates or the free-living community. While no ASV showed significantly different distribution over the entire length of the experiment, several ASVs such as *Rhodobacteraceae* (ASV7), *Alteromonas* (ASV17), *Aestuariibacter* (ASV23), *Pseudooceanicola* (ASV41), and *Thioclava* (ASV77) were more abundant within the microaggregates regardless of treatment during the first 6 days (Fig. 3; Fig. S5). Several ASVs of *Alcanivorax* were statistically more abundant within the aggregated communities but showed different distribution patterns. For example, while *Alcanivorax* ASV13 was identified in every treatment, ASV43 was found only in the oil-amended samples and ASVs 31, 60, and 401 were notably more prevalent in microaggregates only in the oil-plus-dispersant treatments. Conversely, one ASV of *Alcanivorax* (ASV12) was more abundant in the free-living community and found in every treatment. A similar pattern was observed with other groups such as Pseudomonas: ASV1 was preferentially found among the aggregated community while ASV56 was detected in the free-living community. Additionally, some taxa, while detectable at low abundances in all treatments, showed a preference for certain conditions. For instance, ASVs of *Methylophaga* (ASV28 and ASV29) were often associated with microaggregates in the seawater control and oil-only treatments whereas ASVs of *Marinobacter* (ASV65 and ASV240) were predominantly only in the oil-only treatment.

10.1128/mSystems.01105-21.5 FIG S5 Microbial community composition as assessed via PCR amplicon analysis of the V4 region of the 16S rRNA gene. The community’s taxonomic distribution is described as the 20 most abundant genera. Download FIG S5, EPS file, 2.0 MB. Copyright © 2021 Achberger et al. 2021 Achberger et al. https://creativecommons.org/licenses/by/4.0/ This content is distributed under the terms of the Creative Commons Attribution 4.0 International license.

Catalyzed reporter deposition-based fluorescence *in situ* hybridization (CARD-FISH) was used to verify the presence of *Alteromonas* sp. and *Alcanivorax* sp. in the microaggregates as suggested by the 16S rRNA gene analysis (Fig. 2 and Fig. S5). *Alteromonas*-related and *Alcanivorax-*related cells detected by the CARD-FISH probes were most often observed in microaggregate clusters rather than as free-living cells (Fig. S6A). However, there were also several free-living *Alcanivorax* targeted cells identified (Fig. S6B), which is in agreement with their overall distribution in the microbial community data as well as the LDA of ASVs, which confirmed that certain ASVs (e.g., ASV12) were prevalent in the 0.2- to 3.0-μm fraction (Fig. 2 and 3).

10.1128/mSystems.01105-21.6 FIG S6 Catalyzed reporter deposition fluorescence *in situ* hybridization (CARD-FISH) analysis showing the prevalence of targeted cells as either free-living or aggregated. (A) CARD-FISH results for the *Alteromonas*-related species probe. (B) CARD-FISH results for *Alcanivorax*-related species probe. For both panels A and B, the relative abundance of ASVs classified as *Alteromonas* (A) or *Alcanivorax* (B) over time and in the two different size fractions is presented in the left column. The percentage of CARD-FISH-labeled cells present as single, free-living cells or in aggregates is presented in the middle column, and images of representative BOMA from each treatment are presented in the right column. For all micrograph images, CARD-FISH-tagged cells appear green, untagged cells appear blue, and droplets of oil and/or dispersant appear red. Bar, 10 μm. Download FIG S6, EPS file, 9.4 MB. Copyright © 2021 Achberger et al. 2021 Achberger et al. https://creativecommons.org/licenses/by/4.0/ This content is distributed under the terms of the Creative Commons Attribution 4.0 International license.

### Microaggregate communities harbor genes for hydrocarbon degradation.

**FIG 4:**
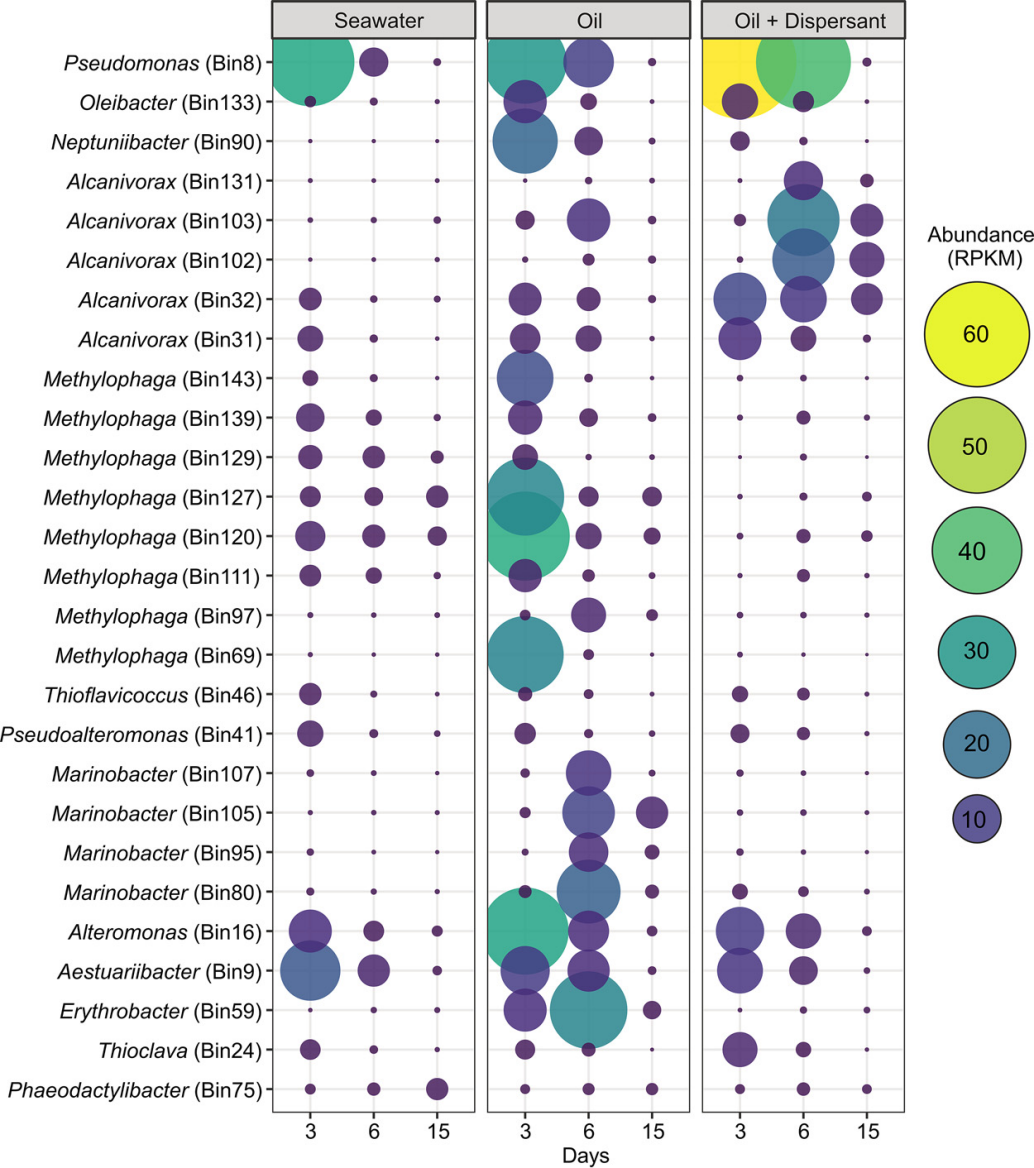
Abundance of metagenome-assembled genomes (MAGs) in microaggregates expressed as reads per kilobase per million (RPKM). Statistics for individual MAGs are provided in Table S1.

Based on the analysis of 16S rRNA genes in combination with hydrocarbon oxidation rates and microaggregate abundance profiles, major shifts in the microaggregate community structure and/or hydrocarbon degradation potential occurred on days 3, 6, and 15. Therefore, these days were chosen for metagenomic analysis of the microaggregate specific community (>3.0 μm) to determine its genetic potential for hydrocarbon oxidation. From these samples, 27 quality metagenome-assembled genomes (MAGs, >50% completion and <10% contamination) were generated that were closely related to bacterial taxa identified as prevalent in microaggregates by the LDA of ASVs (Fig. 4 and Table S1). These MAGs were then searched for genes potentially involved in the degradation of hydrocarbon compounds and their intermediates. The identified genes were consolidated into three groups (alkane, aromatic, and fatty acid degradation; see reference 25). Alkane degradation includes hydroxylase and monooxygenase enzymes which convert alkanes into a fatty alcohol. This alcohol is then further oxidized by alcohol and aldehyde dehydrogenases to a fatty acid and enters the beta oxidation pathway as acyl coenzyme A (acyl-CoA). Because the genes involved in the conversion of the alcohol to a fatty acid and its subsequent breakdown are often involved in metabolisms unrelated to the degradation of alkanes, they were conservatively separated into the fatty acid degradation category. The aromatic degradation category includes enzymes involved in ring hydroxylation and cleavage as well as numerous multistep pathways needed for the continued breakdown of the resultant compounds.

10.1128/mSystems.01105-21.9 TABLE S1 Characteristics of metagenome-assembled genomes presented in this study. Download Table S1, XLSX file, 0.02 MB. Copyright © 2021 Achberger et al. 2021 Achberger et al. https://creativecommons.org/licenses/by/4.0/ This content is distributed under the terms of the Creative Commons Attribution 4.0 International license.

**FIG 5:**
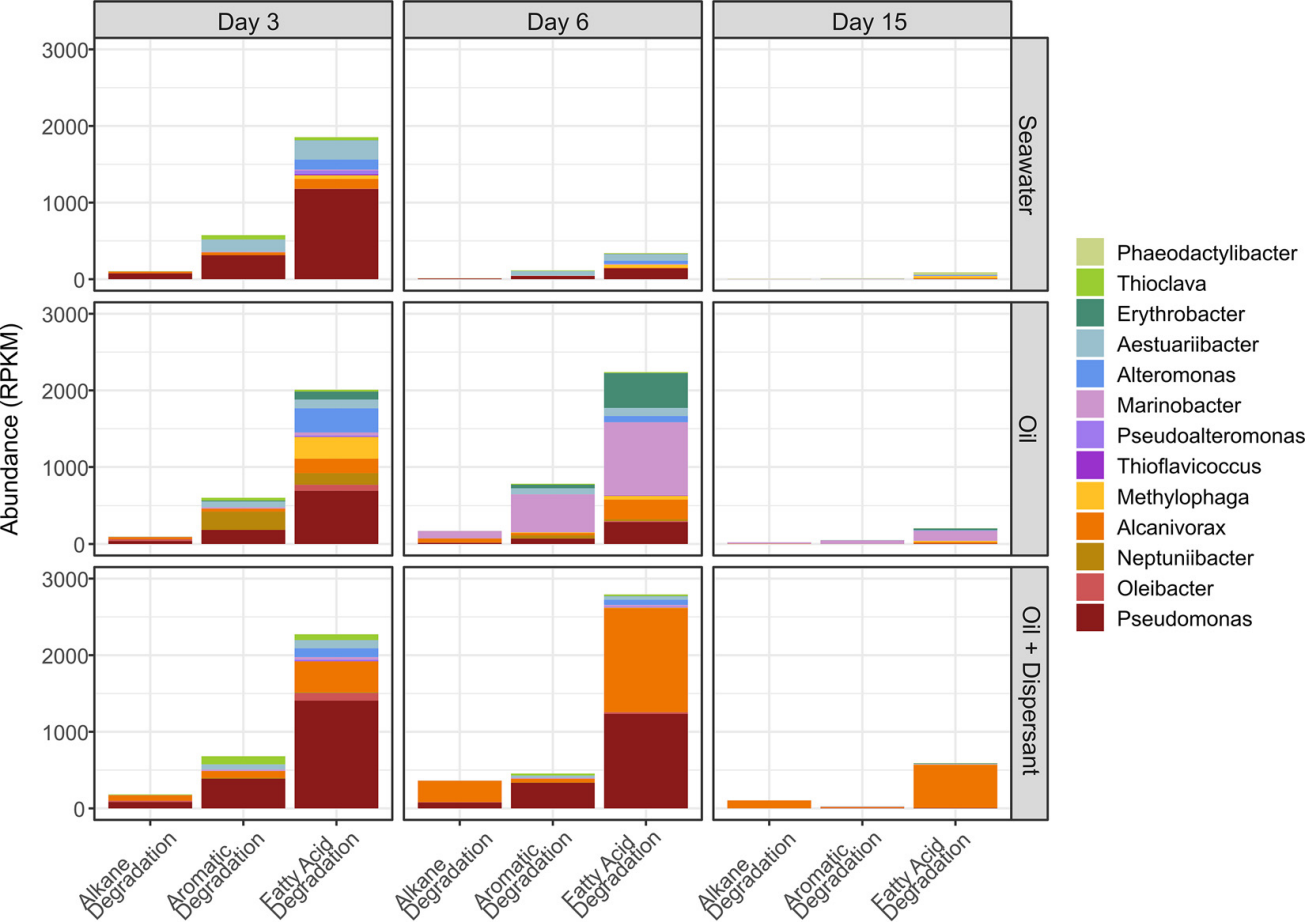
Hydrocarbon degradation potential in metagenome-assembled genomes (MAGs) from taxa more abundant in microaggregates. Abundance of genes involved in the degradation of *n*-alkanes, PAHs, and fatty acids in MAGs.

On day 3 of the experiment, overall abundances of alkane, aromatic hydrocarbon, and fatty acid degradation genes were similar between treatments and largely belonged to a single abundant MAG of Pseudomonas, Bin 8 (Fig. 5). Genes for alkane degradation were also recovered from several MAGs of *Alcanivorax* (Fig. 5). Aromatic hydrocarbon degradation genes belonging to MAGs of *Thioclava* and *Aestuariibacter* were found across all experimental treatments while genes from *Neptuniibacter* were abundant only in the oil-only treatment. As expected, all of these MAGs also contained genes involved in the degradation of fatty acids; however, MAGs of *Alteromonas* and *Methylophaga* appeared to serve a significant function primarily in fatty acid degradation which includes the later steps of alkane oxidation (Fig. 5).

By day 6, there was a notable reduction in hydrocarbon degradation potential in the seawater control treatment. However, the number of hydrocarbon degradation genes in both oil-amended treatments increased from day 3, primarily due to an increased abundance in MAGs of *Marinobacter* and *Erythrobacter* in the oil-only and *Alcanivorax* in the oil-plus-dispersant treatments (Fig. 5). Genes involved in the degradation of alkanes were more numerous in the oil-plus-dispersant treatment compared to the oil-only treatment and were mostly associated with MAGs of *Alcanivorax*. On the other hand, the oil-only treatment had a larger amount of aromatic degradation genes that were primarily related to MAGs of *Marinobacter*. On day 15, when hydrocarbon degradation potential was at its lowest in all treatments, *Marinobacter* and *Alcanivorax* remained the primary hydrocarbon degraders in the two oil-amended treatments.

## DISCUSSION

We sought to evaluate the importance of microaggregates after oil spills and determine if their presence may influence hydrocarbon degradation activity in the water column. Microaggregates have been reported in multiple experimental studies (1, 8–10) and were found in freshly collected surface seawater from the Gulf of Mexico. Although these microaggregates do not need oil to form, in the only previous study where they were quantified, they were shown to be more abundant with increasing oil concentrations (10), and other studies show they often have droplets of oil incorporated into their structure when present (7, 27, 28). However, prior to the current study, whether these BOMAs might play a significant role in the natural biodegradation of spilled oil in the ocean was unexamined.

### BOMA abundance coincides with increased hydrocarbon degradation rates.

Several hydrocarbon-degrading bacteria and eukaryotes produce exopolymeric substances and other biosurfactants in response to the presence of oil as a mechanism to help solubilize and degrade these compounds (7, 15, 29, 30). This can enhance aggregate formation around oil droplets and may have contributed to the large number of BOMAs observed in the oil-amended treatments in this and similar studies. The addition of dispersant which also acts to solubilize hydrocarbons and increases the abundance of smaller oil droplets in the water column may have further amplified this effect (21, 23). Indeed, more BOMAs were formed in the treatment amended with dispersed oil than in that amended with oil alone (Fig. 1A). This trend was also observed in several previous experiments conducted by our research consortium (see Fig. S7 in the supplemental material) (19), indicating that chemical dispersants do not negatively impact the formation of the small-scale aggregates examined in this study but rather emphasize the connection between the abundance of BOMAs and the presence of oil in the water column.

10.1128/mSystems.01105-21.7 FIG S7 Microaggregate abundance as measured in three additional mesocosm experiments. The experimental design from the mesocosms was described previously by Doyle et al. (S. M. Doyle, E. A. Whitaker, V. De Pascuale, T. L. Wade, et al., Front Microbiol 9:689, 2018, https://doi.org/10.3389/fmicb.2018.00689; S. M. Doyle, G. Lin, M. Morales-McDevitt, T. L. Wade, et al., mSystems 5:e00668-20, 2020, https://doi.org/10.1128/mSystems.00668-20). Results from mesocosm 2 were originally presented in the work of Doyle et al. (S. M. Doyle, E. A. Whitaker, V. De Pascuale, T. L. Wade, et al., Front Microbiol 9:689, 2018, https://doi.org/10.3389/fmicb.2018.00689). Results from mesocosms 3 and 4 are previously unpublished. Download FIG S7, EPS file, 2.3 MB. Copyright © 2021 Achberger et al. 2021 Achberger et al. https://creativecommons.org/licenses/by/4.0/ This content is distributed under the terms of the Creative Commons Attribution 4.0 International license.

We utilized a radiotracer-based method to quantify the biological oxidation rates of hexadecane and naphthalene during our experiment. The highest oxidation rates for both compounds occurred in the oil-amended treatments around the same time that the abundance of BOMAs was greatest (e.g., days 3 to 4) (Fig. 1). This evidence supports a linkage between the formation of BOMAs and hydrocarbon degradation. It also indicates that the rates of hydrocarbon degradation in BOMAs may be important in comparison to rates among free-living microorganisms. These findings are the first to link BOMAs to hydrocarbon degradation and are consistent with research showing marine snow and other biomass aggregates are hot spots for microbial activity (28, 31–33).

### BOMAs are distinct from MOS.

While we did find evidence that BOMAs are an important site for hydrocarbon degradation in the marine water column, it remains unclear if BOMAs are a precursor to MOS and the widely reported MOSSFA event that was responsible for transporting up to 31% of oil back to the seafloor following the DwH spill (7, 11–14). Although the number of the microaggregates in our experiment fluctuated over time and with treatment, their overall size did not (Fig. S3). This trend was observed across multiple mesocosm experiments conducted by our research consortium with different seawater sources, nutrient concentrations, and oil/dispersant treatments (Fig. S8) (10, 25, 33). This also suggests BOMAs are a separate phenomenon from larger MOS particles—which in contrast have been demonstrated to coalesce and increase in size over time (28). Our interpretation, however, is complicated by our observation of visible MOS particles cooccurring with microaggregates in our experiments. One possible scenario that resolves this apparent conflict is that BOMAs and MOS particles represent different-size spectra of the same phenomenon. In such a case, our observations indicate a steady-state scenario wherein microaggregates grew larger or stuck together and subsequently sank out over time as MOS while new, smaller microaggregates were formed. However, we cannot rule out that BOMAs and MOS particles are unrelated to one another and BOMAs instead remain small and eventually degrade or disperse into the water column. While both these scenarios merit further study, in either case, BOMAs represent a natural and fundamental component of the microbial response to hydrocarbon exposure which until now has been largely overlooked and underestimated.

10.1128/mSystems.01105-21.8 FIG S8 Size distribution of microaggregates as measured by area in two additional mesocosm experiments. The experimental design for the mesocosms was described previously by Doyle et al. (S. M. Doyle, G. Lin, M. Morales-McDevitt, T. L. Wade, et al., mSystems 5:e00668-20, 2020, https://doi.org/10.1128/mSystems.00668-20). *n* = 90 aggregates per treatment, per time point. Download FIG S8, EPS file, 4.4 MB. Copyright © 2021 Achberger et al. 2021 Achberger et al. https://creativecommons.org/licenses/by/4.0/ This content is distributed under the terms of the Creative Commons Attribution 4.0 International license.

### BOMA are enriched with microorganisms capable of hydrocarbon degradation.

To determine the role BOMAs serve in the biodegradation of oil spills, we utilized size fractionation to specifically target and identify members of the microbial community present in microaggregates and evaluate their potential to degrade hydrocarbons. Within the first few days, the addition of oil and dispersant initiated a rapid growth of putative hydrocarbon-degrading taxa (2, 3). These included the alkane degrader *Oleibacter* (34) and members of the polycyclic aromatic hydrocarbon (PAH)-degrading *Cycloclasticus*, *Neptuniibacter*, and *Alteromonas* genera (35, 36). Also abundant were ASVs of *Marinobacter*, *Alcanivorax*, and Pseudomonas, of which cultured representatives have been shown to degrade a wide range of hydrocarbon compounds (17, 37, 38). All of these taxa were present in the free-living fraction; however, most were shown to be statistically more abundant in the microaggregates based on our analysis of ASVs (Fig. 2 and 3 and Fig. S5). Additionally, most of these taxa have been seen in other microbiological surveys following oil spill events, including that of DwH, and are frequently identified as critical members involved in the natural response to hydrocarbon pollution (1–6). The prevalence of such organisms in BOMAs further highlights the significant role that aggregated microbial consortia likely serve in hydrocarbon degradation.

Analysis of the size-fractionated ASV data implies there is an aggregate-specific microbial community distinct from that found in the background seawater. Highly abundant taxa in seawater such as “*Candidatus* Nitrosopumilus” and OM43 were very rare or absent in the aggregated communities (Fig. S5). Furthermore, individual ASVs of *Alcanivorax* and Pseudomonas exhibited niche separation, showing a distinct preference for either aggregated (ASVs 1, 31, 60, and 401) or free-living (ASVs 12 and 56) lifestyles. It should also be noted that many of the taxa present in BOMAs (e.g., *Alcanivorax*, *Marinobacter*, Pseudomonas, *Pseudoalteromonas*, and *Alteromonas*) have been shown to produce exopolymeric substances and associated biosurfactants in the presence of oil, which promotes aggregation (4, 13, 39). Together, this suggests that BOMAs are formed and maintained by a selective group of microorganisms.

Of those taxa that were found to be prevalent in microaggregates in our ASV analysis (Fig. 3), many showed the genetic potential to degrade various hydrocarbon compounds based on a metagenomic analysis of the >3.0-μm size fraction (Fig. 5). Early in the experiment (day 3), all treatments showed comparable abundances of hydrocarbon degradation genes, including the seawater control. This was not unexpected given that oil is continually introduced into the Gulf of Mexico through natural seeps and spills of various sizes (40), and putative hydrocarbon-degrading microorganisms (e.g., Pseudomonas, *Alteromonas*, *Alcanivorax*, and *Rhodobacteraceae*) were abundant in the freshly collected seawater used to establish the mesocosms. However, without the addition of new oil, the abundance of hydrocarbon degradation genes rapidly declined in the seawater control treatment.

We detected a higher abundance of genes needed for the degradation of aromatic hydrocarbons in the oil-only treatment than in oil plus dispersant. This is in agreement with the higher rates of naphthalene oxidation measured in the oil-only treatment and with the compositional analysis of individual hydrocarbon compounds in our mesocosms, which revealed that the concentration of total PAHs, including naphthalene, was also higher in the oil-only samples (24). The majority of these genes were recovered from MAGs of *Marinobacter* and *Neptuniibacter*, PAH-degrading taxa that showed a preference for the oil-only treatment (Fig. 4; Fig. S5). Conversely, genes involved in the degradation of alkanes were more abundant in the oil-plus-dispersant treatment and largely associated with *Alcanivorax* and Pseudomonas. These data are again in agreement with the slightly higher concentrations of total *n*-alkanes measured at the start of the experiment in the oil-plus-dispersant treatment than in oil alone (24). Overall, these results indicate that the changes in the structure of the BOMA community, and thus their ability to degrade specific hydrocarbons, were shaped by the initial availability of individual hydrocarbon compounds (e.g., *n*-alkanes versus PAHs). This variability observed in the composition of the hydrocarbons between the oil-only and oil-plus-dispersant treatments may be attributed to the addition of dispersant, which has been shown to influence the amount of different hydrocarbon compounds accommodated into seawater (24, 41) as well as the biological outcome (9, 10).

In the second half of our experiment, as the total hydrocarbon concentrations and rates of oxidation decreased (Fig. 1), so too did the abundance of BOMAs and hydrocarbon-degrading microorganisms. These taxa were replaced by members of *Flavobacteriaceae*, *Roseibacillus*, *Loktanella*, *Pseudohongiella*, and *Ruegeria*, many of which exhibit heterotrophic lifestyles not specifically associated with oil degradation (Fig. 2 and Fig. S5) (42–45). Such organisms may be utilizing the degradation products of the hydrocarbons (29), the nutrient-rich EPS of the aggregates (32), or other cellular components. Indeed, high rates of polysaccharide degradation by heterotrophic species following the formation of oil aggregates have been observed in several studies (e.g., references 31 and 32) and were also observed during this experiment (33). This shift toward secondary consumers marks a successional cascade in the aggregated microbial community following the initial degradation of hydrocarbons and may serve as an indicator of oil spill recovery.

### Conclusion.

The large oil-associated aggregates that formed following the DwH spill event were identified as a key factor influencing the transport of hydrocarbons from surface waters to the seafloor. However, the potential for these aggregated microorganisms to influence the degradation of the incorporated oil droplets was poorly understood. The evidence presented in this study reveals that bacterially dominated micrometer-scale aggregates are enriched in hydrocarbon-degrading taxa and primed to utilize a wide range of hydrocarbon compounds. Although it remains unknown if the BOMAs studied here eventually coalesce with time and seed MOS formation, the work presented here indicates BOMAs are an important and fundamental aspect of how microorganisms respond to marine oil spills and provides a previously overlooked mechanism for hydrocarbon degradation in the water column. Given their abundance following oil spills, it is possible that BOMAs also occur in other environments where hydrocarbons are released into the water column, such as at methane seeps and hydrothermal vents. Consequently, the efficiency with which such aggregated microbial consortia degrade hydrocarbons or certain components of oil compared to free-living cells is critical to determining the lifetime of hydrocarbons in the ocean and remains unconstrained.

## MATERIALS AND METHODS

### Experimental design.

In May 2017, surface seawater was collected from the coastal Northern Gulf of Mexico (29°27 N; 94°81 W) and used to establish a mesocosm-scale experiment (100 liters) with three treatments: (i) natural seawater, (ii) seawater amended with Macondo surrogate crude oil (oil-only), and (iii) seawater amended with chemically dispersed oil (oil plus dispersant; see reference 33). The oil treatment was generated by mixing 25 ml of Macondo surrogate crude oil with 130 liters freshly collected seawater for 4 h using a 170-liter baffled recirculating tank (46). This material was then divided into three 100-liter replicate tanks. To generate a chemically dispersed oil, 25 ml of Macondo surrogate crude oil and Corexit (20:1) was added to seawater and mixed as described above. This material was then divided and subsequently diluted 10-fold among three tanks to create the oil-plus-dispersant treatment that had oil concentrations consistent with those found in the oil-only treatment as well as those observed during the Deepwater Horizon oil spill event (46). Immediately prior to the start of the experiment, a freshly collected natural phytoplankton mixture was added to each of the tanks (starting biomass, ∼2 to 4 μg/liter). Details on nutrient concentrations are provided elsewhere (33). Tanks were maintained for 16 days at room temperature on a 12-h light/dark cycle, with 50 to 60 μmol photons m^−2^ s^−1^, and samples were collected into 1-liter polycarbonate bottles from a spigot on the mesocosm tank.

### Estimated oil equivalents.

Every 24 h, 5 to 20 ml of seawater was collected from the spigot and extracted with dichloromethane (DCM) according to the method of Wade et al. (47), as updated in the work of Wade et al. (46). DCM-extractable hydrocarbons (3 ml) were analyzed using a Horiba Aqualog-UV-800 to measure fluorescence at an excitation of 260 nm and an emission of 372 nm. Fluorescence intensity was compared to a standard curve generated from Macondo surrogate oil to determine the estimated oil equivalent (EOE) concentration. At these wavelengths, chlorophyll *a* (chl *a*) and accessory pigments, as well as colored dissolved organic matter, may generate artificially elevated values, so additional controls were used to correct EOEs. Student’s *t* test was used to determine the significances of differences in oil concentrations between treatments. Additional details about the hydrocarbon composition and degradation rates are provided elsewhere (24).

### Hydrocarbon oxidation rate.

Rates of biological hydrocarbon oxidation were estimated using [1-^14^C]hexadecane and [1-^14^C]naphthalene (American Radiolabeled Chemicals Inc.). During each time point tested, duplicate 1-ml water samples were collected from each of the triplicate mesocosm tanks for each of the three treatments (seawater control, oil-only, and oil-plus-dispersant). To each sample, 100,000 dpm of either ^14^C-labeled hexadecane or naphthalene was added. The samples were incubated in an airtight vial with a carbon dioxide trap consisting of 100 ml of 0.5 mol liter^−1^ KOH for 12 to 24 h before being terminated with the addition of 50 μl of 100% trichloroacetic acid (TCA) injected through the silicone septum cap. The samples were allowed to sit for another 6 h before the traps were removed to allow for maximum absorbance of liberated ^14^CO_2_. The contents of the traps were transferred to a clean 20-ml scintillation vial, and 10 ml of CytoScint ES (MP Biomedicals) scintillation cocktail was added. Counts were measured for 3 min on a Beckman Coulter liquid scintillation counter. Killed controls were periodically tested throughout the experiment by either pasteurizing the sample at 80°C for 1 h or acidifying the sample to pH ≤2 prior to the addition of radiolabeled substrate and incubation. Background incorporation detected in controls was subtracted from rates measured in samples. Samples were collected every 24 h for the first 4 days and then every 48 h until the end of the experiment. Student’s *t* test was used to determine the significances of differences in rates between treatments.

### Microscopy and image analysis.

To monitor the number of microbial cells and the formation of micrometer-scale aggregates, water was collected from the freshly collected seawater used to establish the mesocosms and from each tank at each time point and preserved with either formalin (2% final concentration) for standard cell counts or paraformaldehyde (2% final concentration) for catalyzed reporter deposition fluorescence *in situ* hybridization (CARD-FISH). The formalin-preserved samples were processed for cell and microaggregate counts as described previously (10). Briefly, samples were stained with 4′,6-diamidino-2-phenylindole (DAPI; 45 μM) nucleic acid dye for 10 min and filter concentrated under vacuum onto 0.2-μm-pore-size black polycarbonate filters. Filters were then mounted on a glass microscope slide using CitiFluor AF1 antifading mounting solution. The total number of DAPI-stained cells in 10 fields of view was counted using ×1,000 magnification on a Zeiss fluorescence microscope (Axio Imager M2; Zeiss, Jena, Germany). To quantify the number of cell aggregates in each of the treatments, total aggregates were counted at ×400 magnification in 10 fields of view per sample on the same DAPI-stained slide used for total cell enumeration. We defined an aggregate as a tight cluster of 10 or more individual cells. These counts are conservative estimates for the number of microaggregates considering it is possible that some microaggregates may have overlapped during filtration and/or slide preparation. We did not observe evidence of this during our counting; however, if it were to have occurred, it would result in fewer microaggregates being counted than were present. A Student *t* test was used to determine the significances of differences in cell and aggregate abundances between treatments.

To determine aggregate size, images were taken of 100 aggregates per treatment per time point tested and the area of each aggregate was manually calculated using ImageJ software. Differences in aggregate size distribution were evaluated using a Tukey *post hoc* analysis.

CARD-FISH samples were incubated at 4°C for 12 to 16 h before being filter concentrated onto a 0.2-μm-pore-size white polycarbonate filter and washed twice with 1 volume of phosphate-buffered saline (137 nM NaCl, 2.7 nM KCl, 10 mM Na_2_HPO_4_, 1.8 mM KH_2_PO_4_). The filters were allowed to dry completely and stored at −20°C. CARD-FISH was performed following the protocols outlined in the work of Pernthaler et al. (48) and Pernthaler and Pernthaler (49). Horseradish peroxidase-modified probe ALT1413 (5′-TTTGCATCCCACTCCCAT) was used to target *Alteromonas*- and *Colwellia*-related species and hybridized with a 40% formamide concentration. A second probe, ALC665 (5′-CGGAAATTCCACCTCCCT), was designed using the Probe Design function in ARB against the SILVA v132 small-subunit database to target the most abundant *Alcanivorax* ASV present in this study. The probe specificity was checked with the SILVA probe match and evaluation tool (TestProbe), and the optimum formamide concentration for hybridization (55%) was determined empirically using isolate LSUCC0630 cultured from the northern Gulf of Mexico (50). Alexa Fluor 488-conjugated tyramide was used to visualize probe deposition, and all cells were counterstained with DAPI. The proportion of probe-targeted cells to total cells was determined in 10 fields of view per sample.

### LSUCC0630 isolate cultivation.

Isolate LSUCC0630 was obtained from the LSUCC culture collection. It was isolated from source water collected on a research cruise in the Gulf of Mexico (PE17-02, Lead Chief Scientist Frank Stewart) from Station C6C (lat 28.8687, long −90.4785) on 2 August 2016 using a Niskin rosette onboard the RV *Pelican*. The sample was collected from the Niskin bottle at 14 m (temperature 27.32°C, salinity 35.31). For collection, a 1-liter acid-washed and autoclaved Pyrex bottle was filled with Niskin water until overflowing and sealed. Isolation was performed in custom medium according to the work of Henson et al., 2016 (50).

### Molecular biology analysis.

Water samples (200 ml) were collected daily from each tank and first gravity filtered through a 47-mm-diameter, 3.0-μm-pore-size Isopore TSTP filter to collect microaggregates. The filtrate was subsequently filtered under vacuum onto a 47-mm-diameter, 0.2-μm-pore-size Supor membrane filter to collect free-living cells. Additional water samples were collected from the starting seawater and from each of the oil and oil-plus-dispersant mixing tanks to account for any changes in the microbial community that may have occurred during experimental setup. These samples (200 ml) were filtered directly onto a 47-mm, 0.2 -μm Supor membrane filter with no prefiltration. All filters were placed into cryovials and immediately preserved at −80°C until extraction. DNA was extracted from each filter using the FastDNA spin kit for soil (MP Biomedicals) according to the manufacturer’s protocol.

For microbial community analysis, the V4 hypervariable region of the 16S rRNA gene was amplified using universal primers 515F and 806R (10, 51) and sequenced on the Illumina MiSeq platform (500 cycle, 250-bp paired end [PE]) at the Georgia Genomics Facility (Athens, GA, USA). Raw 16S rRNA gene sequence reads were curated and coalesced into amplicon sequence variants (ASVs) using the standard pipeline of the DADA2 R package (52). All ASVs were subsampled to an even depth before downstream ecological analyses were performed with mothur (53) or the R packages phyloseq (54) and vegan (55). Changes in microbial community structure between treatments and over time were examined using nonmetric multidimensional scaling (NMDS) based on a Bray-Curtis dissimilarity index and tested for significance using analysis of molecular variance (AMOVA) (56). To evaluate which individual ASVs differentiated the microbial communities found on the 0.2- to 3.0-μm and >3.0-μm filter size fractions in each treatment and at each time point, a linear discriminant analysis (LDA) combined with effect size measurements (26) was used. Significance of differential abundance was based on a *P* value of <0.05 in the Kruskal-Wallis test and a score of ≥2.0 in pairwise Wilcoxon tests.

For metagenomic analysis, DNA extracted from the 3.0-μm filters from three representative time points (days 3, 6, and 15) was sequenced on the Illumina HiSeq platform (100-bp PE) at the University of Delaware DNA Sequencing & Genotyping Center (Newark, DE, USA). DNA from triplicate tanks was pooled in equimolar concentration and treated as a single sample for sequencing. Raw reads were trimmed and filtered for quality using Trimmomatic (average quality = 30, minimum length = 75) (57). All samples were pooled, assembled with IDBA-UD (58), and binned with MaxBin2 (59) using default parameters. Individual sequence reads from each sample were then mapped to the binned contigs using Bowtie2 (60) to determine coverage across samples. Bins were imported into Anvi’o (61) where genes were identified, annotated according to the Kyoto Encyclopedia of Genes and Genomes (KEGG) database, and taxonomically assigned using Kaiju (62) by comparison to the NCBI nr database. Bins were evaluated for completeness and contamination using CheckM (63) and manually refined in Anvi’o. Curated bins with >50% completeness and <10% contamination were retained for further analysis (see Table S1 in the supplemental material). The abundance of bins and genes across samples was determined by evaluating the mapped reads using RPKM (reads per kilobase per million) normalization with the formulation: *Aij* = (*Nij*/*Li*) × (1/*Tj*), where *Aij* is the abundance of bin *i* in sample *j*, *Nij* is the number of reads that map to bin *i* from sample *j*, *Li* is the length of bin *i* in kilobases, and *Tj* is the total number of reads in sample *j* divided by 10^6^ as described in reference 64. Genes belonging to a KEGG pathway involved in the degradation of saturated hydrocarbons (alkanes and cycloalkanes), PAHs, or their metabolic intermediates in central metabolism (glycolysis, tricarboxylic acid cycle) were specifically identified and quantified (Table S2) (26, 65).

10.1128/mSystems.01105-21.10 TABLE S2 List of genes involved in KEGG pathways associated with the degradation of various hydrocarbon compounds (see S. M. Doyle, G. Lin, M. Morales-McDevitt, T. L. Wade, et al., mSystems 5:e00668-20, 2020, https://doi.org/10.1128/mSystems.00668-20). Download Table S2, XLSX file, 0.02 MB. Copyright © 2021 Achberger et al. 2021 Achberger et al. https://creativecommons.org/licenses/by/4.0/ This content is distributed under the terms of the Creative Commons Attribution 4.0 International license.

### Data availability.

Sequences were submitted to the National Center for Biotechnology Information Sequence Read Archive under BioProject PRJNA629337 (metagenomes) and BioProject PRJNA623576 (16S rRNA amplicons). Data are publicly available through the Gulf of Mexico Research Initiative Information and Data Cooperative (GRIIDC) at http://data.gulfresearchinitiative.org (DOIs: https://doi.org/10.7266/3ZVPZ2F8, https://doi.org/10.7266/n7-z8x6-km14, https://doi.org/10.7266/N71GNKT1, https://doi.org/10.7266/n7-v9q1-5116, and https://doi.org/10.7266/n7-1kx8-0z36).
